# Validation of spline modeling for calculation of electron insert factors for varian linear accelerators

**DOI:** 10.1002/acm2.13430

**Published:** 2021-10-05

**Authors:** Garrett C. Baltz, Steven M. Kirsner

**Affiliations:** ^1^ Scripps MD Anderson Cancer Center San Diego California USA

## Abstract

There are several methods available in the literature for predicting the insert factor for clinical electron beams. The purpose of this work was to build on a previously published technique that uses a bivariate spline model generated from elliptically parameterized empirical measurements. The technique has been previously validated for Elekta linear accelerators for limited clinical electron setups. The same model is applied to Varian machines to test its efficacy for use with these linear accelerators. Insert factors for specifically designed elliptical cutouts were measured to create spline models for 6, 9, 12, 16, and 20 MeV electron energies for four different cone sizes at source‐to‐surface distances (SSD) of 100, 105, and 110 cm. Insert factor validation measurements of patient cutouts and clinical standard cutouts were acquired to compare to model predictions. Agreement between predicted insert factors and validation measurements averaged 0.8% over all energies, cones, and clinical SSDs, with an uncertainty of 0.6% (1SD), and maximum deviation of 2.1%. The model demonstrated accurate predictions of insert factors using the minimum required amount of input data for small cones, with more input measurements required for larger cones. The results of this study provide expanded validation of this technique to predict insert factors for all energies, cones, and SSDs that would be used in most clinical situations. This level of accuracy and the ease of creating the model necessary for the insert factor predictions demonstrate its acceptability to use clinically for Varian machines.

## 1 INTRODUCTION

There are many methods in the literature for determination of the insert factor required for calculating the dose delivered by a clinical electron beam. Circular or rectangular shaped fields have established analytical formulas that can be used to calculate the insert factor.^1^, ^2^, ^3^, ^4^ Irregularly shaped electron fields are more common for electron treatments and present additional challenges for insert factor determination. Radiotherapy clinics that rarely treat with electrons often choose to measure the insert factor for each unique electron field.^5^ This method can be laborious and time prohibitive for larger clinics that frequently use electron fields. As an alternative, several techniques for the analytical calculation of the insert factor for irregularly shaped insert factors have been developed.^6^, ^7^, ^8^, ^9^, ^10^, ^11^ The expected accuracy to calculate insert factors utilizing these techniques ranges from 1.0% to 5.9%.^12^ Newer treatment planning systems which can model electron beams using Monte Carlo methods are able to determine the beam output directly via the physics simulation^13^, ^14^, ^15^; however it is still best practice to verify the output with a secondary monitor unit (MU) calculation.^16^

One such analytical technique presented by Biggs et al. allows for the calculation of the insert factor for any irregular shape using a bivariate spline model fit to empirically measured output factors.^17^ This technique relies on parameterizing insert shapes into equivalent ellipses, characterized by the width and perimeter‐to‐area ratio of the insert. Parameterizing insert shapes into ellipses was found to be correlated with the primary factors that affect change in electron beam output by the insert, including changes in lateral scatter, bremsstrahlung produced in the insert material, and scatter from the edge of the insert. A two‐dimensional parameter space of measured insert output factors as a function of parameterized width and perimeter‐to‐area ratio is used to fit a bivariate spline, from which the output factor for an irregular insert shape can be interpolated.

Biggs et al. validated their technique for a 10 cm electron applicator for an Elekta Agility (Stockholm, Sweden) linear accelerator. The purpose of the current study was to validate the ability of a bivariate spline model to analytically calculate the insert factor for irregularly shaped fields across the range of electron energies and cone sizes for the Varian (Palo Alto, California) Clinac iX and TrueBeam linear accelerators.

## 2 METHODS

### 2.1 Definition of insert factor

In our clinic, the MUs for a clinical electron field are calculated using Equation 1.

(1) $$\mathrm{MU}=\frac{\mathrm{Dose}\;(\mathrm{cGy})}{\begin{array}{c} \left(1\;\frac{\mathrm{cGy}}{\mathrm{MU}}\right)\times \left(\mathrm{Pres}\mathrm{crib}\mathrm{ed}\;\mathrm{Isod}\mathrm{ose}\;\mathrm{Line}\right)\\ \times \;(\mathrm{Cone}\;\mathrm{Fact}\mathrm{or})\times (\mathrm{Inse}\mathrm{rt}\;\mathrm{Fact}\mathrm{or}) \end{array}}$$

Where:

(2) $$Cone\;Factor=\frac{{\left(Output\;Open\;Treatment\;Cone\right)}_{Treatment\;SSD}}{{\left(Output\;Open\;15\times 15\;Cone\right)}_{100\;SSD}}$$

(3) $$Insert\;Factor=\frac{{\left(Output\;Insert\right)}_{Treatment\;SSD}}{{\left(Output\;Open\;Cone\right)}_{Treatment\;SSD}}$$

Equation 3 is the definition used for insert factor in this study.

### 2.2 Model data collection

Empirically measured insert factors for known shapes are used as the basis to create the spline model used to calculate the insert factor for any given irregularly shaped insert. The order of the spline function used requires a minimum of eight data points to create a model. To maximize the ability of the spline model to accurately calculate the insert factor across the full range of insert shapes possible for a given cone size, while also minimizing the number of measurements required, Biggs et al. recommends taking measurements of shapes that bound the range of possible equivalent ellipses that can fit in a given cone. This can be accomplished by measuring multiple ellipses with a length equal to the maximum diagonal dimension of the cone with varying widths, combined with varying diameter circular shapes which represent an ellipse having equal length and width. The insert shapes designed to meet these criteria are illustrated in Table 1. For the larger cones, additional shapes beyond the minimum eight were acquired to increase model robustness.

**TABLE 1:** Insert shapes measured for 6, 10, 15, and 20 cm cones

A06 cone	A10 cone	A15 cone	A20 cone
2.5 × 7 ellipse	2.5 × 13 ellipse	2.5 × 19 ellipse	3 × 21 ellipse
4 × 6.5 ellipse	4 × 12 ellipse	4 × 19 ellipse	6 × 21 ellipse
4 × 5 ellipse	7 × 11 ellipse	7.5 × 18 ellipse	9 × 21 ellipse
2.5 × 3 ellipse	5 × 10 ellipse	10 × 17 ellipse	5 × 17 rectangle
5.5 circle	4 × 8 ellipse	7 × 12 ellipse	3 × 10 rectangle
4 circle	3 × 5.5 ellipse	6 × 8 ellipse	19 circle
3 circle	9.5 circle	14.5 circle	15 circle
2.5 circle	8 circle	12 circle	11 circle
	6 circle	9 circle	8 circle
	4 × 4 square	6 circle	11 × 20 rectangle
		4 × 4 square	

Each insert shape was created in the Varian Eclipse treatment planning system, version 15.6, and a printout of the beams eye view at 100 SSD was used to cut the Cerrobend insert with the Aktina (Congers, NY) electron beam cutting and shaping system.

The insert factor varies with energy, cone, and SSD. Therefore, a spline model must be created for each electron energy, cone, and SSD combination that could be used clinically, requiring insert factors to be measured for each combination. In our clinic, treatments use electron energies of 6, 9, 12, 16, and 20 MeV with SSDs of 100, 105, or 110 cm.

The depth of maximum dose (d_max_) for each insert shape must be determined to accurately measure the insert factor. Hence, for each of the inserts, a percent depth dose (PDD) scan was performed to determine the actual d_max_ for each insert and electron energy. Percent depth ionization (PDI) scans were acquired at 100 cm SSD with the Sun Nuclear (Melbourne, Fl) 3DS scanning system using a Sun Nuclear 0.125cc ionization chamber. The PDI scans were then converted to PDD using the AAPM TG‐70 protocol in the Sun Nuclear Dosimetry software. The d_max_ obtained from the PDD scans for each insert was recorded and used for the subsequent ionization measurements. Since the PDD for electron beams does not vary strongly with a change in SSD,^18^ the same d_max_ was used for all SSDs.

To determine the insert factor, output measurements at d_max_ were acquired for both the insert and the open cone. Measurements were acquired utilizing the same scanning system and with the same ion chamber at the effective point of measurement. The effective point of measurement was calculated using Equation 2 in AAPM TG‐25.^18^ Ionization readings for each insert were acquired by delivering 100 MUs multiple times and then taking the average as the final ionization reading to be used. Insert factors were calculated by taking the ratio of the reading for the insert to that of the open cone, as demonstrated in Equation 3. When there was a change in d_max_ for the insert measurement versus the open cone, the ionization readings were corrected for the change in stopping power ratio based on Table 2 in TG‐25.^18^

This procedure was used to measure the insert factors for every insert listed in Table 1, for 6, 9, 12, 16, and 20 MeV, and at SSDs of 100, 105, and 110 cm. These same set of measurements were acquired on both a Varian iX as well as a Varian TrueBeam linear accelerator. This allowed for the creation of spline models to predict insert factors for all possible electron treatments on either machine.

### 2.3 Software development for model implementation

A web‐based Python (version 3.7.9) application was developed to generate the spline model and calculate the insert factor for an irregular insert. The code used to parameterize insert shapes and generate the spline model was the electron factors module in the open‐source Python package PyMedPhys version 0.33.0 made available by Biggs et al.^19^

The RT Plan DICOM data created in Eclipse for every insert shape measured in Table 1 were exported and used to extract the insert shape and determine the shape's equivalent ellipse. A shape's equivalent ellipse is parametrized by the width and length, where the width of the ellipse is determined by the diameter of the largest circle fully enclosed by the insert shape, and the ellipse length is set to a value such that the area of the ellipse is equal to that of the insert shape. A database text file was created which contains the insert parameterized shape data and corresponding measured insert factors.

To calculate the insert factor for an arbitrary shape, the program reads in an RT Plan DICOM file exported from Eclipse, from which the beam parameters and insert shape are extracted, and then uses the database text file to generate a spline model for the cone, energy, and SSD combination that matches that of the insert. The generated spline model is then used to calculate the insert factor. Finally, the application provides a report of the beam parameters, insert shape, shape parameterization, and the calculated insert factor.

### 2.4 Model validation

To validate the model, insert factor measurements were performed for a sampling of patient inserts that were under treatment, as well as a variety of standard circle and ellipsoid inserts that are used for treatments. A broad range of inserts sizes, beam energies, and SSDs were chosen to validate the model's ability to accurately calculate insert factors across the full range that would be used clinically.

Insert factors for the validation inserts were acquired using the same process described in Section 2.2. Patient validation measurements were acquired on a Varian iX, while the standard insert validation measurements were acquired on a Varian TrueBeam.

**FIGURE 1:**
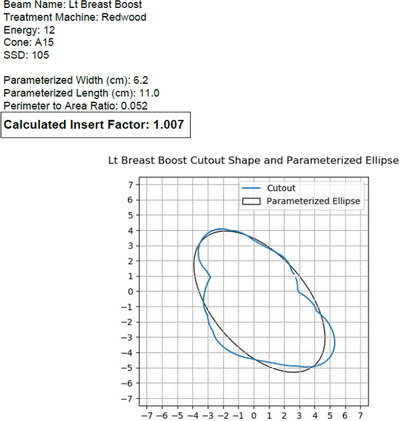
Sample output of insert factor calculation program

To calculate the insert factors, the clinically used DICOM RT plan data for each patient and each standard insert were exported from Eclipse to the web‐based insert factor program detailed in Section 2.3. The program was run for each patient and each standard insert, and a PDF report was saved of the parameterization and calculated insert factor. An example of the report is presented in Figure 1. The calculated insert factors were then compared to the measured values.

## 3 RESULTS

Tables 2 and 3 list all the validation measurements performed, including the relevant field parameters, estimated insert dimensions, measured insert factor, program predicted insert factor, and the percent difference.

**TABLE 2:** Results of patient insert validation measurements acquired on Varian iX

Patient #	Energy	Cone	SSD	d_max_ (cm)	Estimated dimensions (cm)	Measured insert factor	Calculated insert factor	Percent difference
1	6	6	110	1.3	3.5 × 5.5 ellipse	0.884	0.892	0.9%
2	6	6	105	1.3	3.5 × 4.5 ellipse	0.963	0.982	2.0%
3	6	10	110	1.3	7 cm circle	0.984	0.996	1.2%
4	6	10	105	1.3	6 × 8 ellipse	0.993	1.002	0.9%
5	6	15	105	1.2	10 × 8 ellipse	1.007	1.008	0.1%
6	9	6	105	1.7	3 × 4 ellipse	0.899	0.898	−0.1%
7	9	6	110	1.8	4 cm circle	0.901	0.901	0.0%
8	9	10	105	2.0	5 × 6 ellipse	0.980	0.995	1.5%
9	9	10	105	2.0	5 × 8.5 ellipse	0.984	1.000	1.6%
10	9	15	105	2.0	12 × 9 ellipse	1.008	1.006	−0.2%
11	9	15	110	2.0	6 × 9 ellipse	0.992	1.003	1.1%
12	9	15	110	2.0	4 × 7 ellipse	0.949	0.966	1.8%
13	9	20	110	2.0	9 × 17 ellipse	1.006	1.002	−0.4%
14	12	10	105	2.8	8 × 7 ellipse	0.995	1.001	0.6%
15	12	10	110	2.4	4 × 7.5 ellipse	0.902	0.915	1.4%
16	12	15	105	2.4	4 × 8 ellipse	0.970	0.971	0.1%
17	12	15	105	2.7	6 × 9 ellipse	1.000	1.007	0.7%
18	12	15	105	2.8	10 cm circle	1.002	1.006	0.4%
19	12	20	110	2.8	5 × 17 ellipse	0.961	0.966	0.5%
20	16	10	105	3.2	8 × 7 ellipse	0.989	1.000	1.1%
21	16	10	110	2.8	4 × 7.5 ellipse	0.934	0.953	2.1%
22	20	10	105	2.0	3.5 × 8 ellipse	0.974	0.992	1.8%

**TABLE 3:** Results of standard insert validation measurements acquired on Varian TrueBeam

Cutout	Energy	Cone	SSD	d_max_ (cm)	Measured insert factor	Calculated insert factor	Percent difference
3 × 4	6	6	100	1.2	0.935	0.946	1.2%
4.5 × 3.5	6	6	100	1.3	0.979	0.975	−0.4%
3 × 3.5	6	6	100	1.1	0.924	0.936	1.3%
C5	6	6	105	1.3	0.986	0.98	−0.6%
6 × 5	6	10	105	1.2	0.995	0.983	−1.2%
6 × 8	6	10	110	1.2	0.986	0.984	−0.2%
6 × 12	6	15	110	1.2	0.981	0.981	0.0%
4 × 17	6	20	100	1.2	0.987	0.975	−1.2%
4 × 17	6	20	110	1.2	0.898	0.887	−1.2%
C5	9	6	100	2.0	0.982	0.98	−0.2%
4.5 × 3.5	9	6	105	1.8	0.944	0.94	−0.4%
3 × 3.5	9	6	105	1.5	0.865	0.870	0.7%
7 × 9	9	10	100	2.0	1.006	1.006	0.0%
C7	9	10	105	2.0	0.999	0.994	−0.5%
C10	9	15	105	2.0	1.006	0.999	−0.7%
7 × 19	9	20	105	2.0	0.996	0.996	0.0%
5 × 8	12	10	105	2.7	0.983	0.983	0.0%
8 × 14	12	15	110	2.8	1.001	1.007	0.6%
7 × 10	12	10	105	2.8	1.000	1.006	0.6%
C7	12	10	100	2.8	0.998	0.994	−0.4%
6 × 10	16	10	100	2.8	1.005	1.001	−0.4%
7 × 9	16	10	105	3.2	1.001	1.000	−0.1%
7 × 14	16	15	100	3.2	1.008	1.018	1.0%
9 × 19	16	20	100	3.2	1.013	1.015	0.2%
6 × 8	20	10	105	2.0	1.001	0.997	−0.4%
8 × 14	20	15	110	2.8	1.000	1.006	0.6%

For the patient cases, the average absolute percent difference between measured and calculated insert factors was 0.9% with a maximum deviation of 2.1% and a standard deviation of 0.8% (1SD). A possible limitation of the validation measurements, which may have led to larger discrepancies between the program's calculations and measurements, is that some of the inserts were not centered about the central axis.

For the standard insert validation, the average absolute percent difference between calculated and measured values was 0.6%. The maximum deviation for any case was 1.9%. The standard deviation was 0.8%.

**FIGURE 2:**
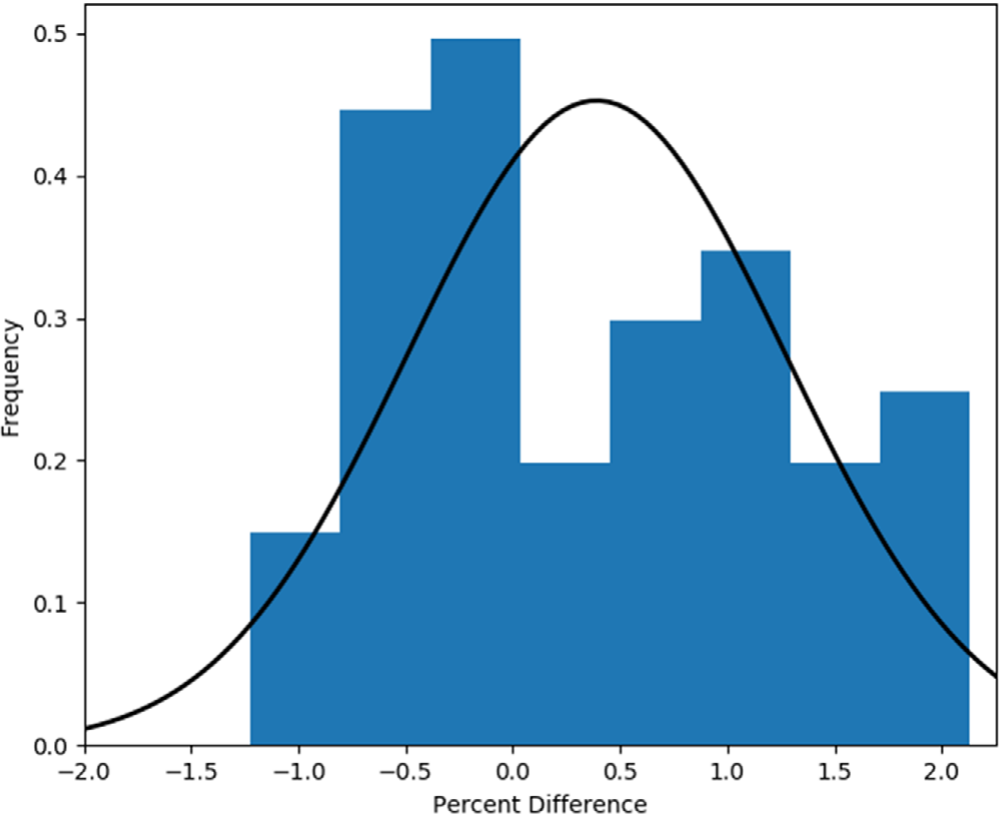
Histogram of percent difference for all validation insert factor measurements. The Gaussian fit is overlayed with parameters *μ* = 0.4% and *σ* = 0.8%

The combination of all 42 insert validation comparisons yields a mean absolute difference of 0.8% with a maximum difference of 2.1% and a standard deviation of 0.6%. A histogram illustrating the distribution of the agreement between predicted and measured insert factors is presented in Figure 2. A Gaussian was fit to the data considering the distribution of positive and negative predictions, which yielded a mean of 0.4% and standard deviation of 0.9%.

## 4 DISCUSSION

The accuracy for the calculation of an insert factor for an arbitrary insert shape demonstrated by the current study compares similarly to previous methods presented in the literature. One dimensional methodology demonstrates an accuracy between measured and calculated insert factors of 2%–3%, pencil beam methods 2%–2.7%, and sector integration methods ranges from 1% to 3%.^12^ The validation measurements performed in this study yielded an absolute percent difference of 0.8%, with a maximum deviation of 2.1%. Biggs et al. reported an average percent difference of 0% for their validation measurements, due to considering the sign of the difference and predictions being equally distributed from ‐1.0% to 1.0%. For the current study, the average percent difference calculated considering the sign was 0.4%. This demonstrated the models for this study have a slight bias toward the predicted insert factors being larger than measured.

The results of the current study provide expanded validation on the efficacy of using the bivariate spline model with elliptical shape parameterization for the calculation of insert factors. Biggs et al. validated the technique for 6, 9, 12, 15, and 18 MeV electrons with a 10 cm cone at 100 SSD for an Elekta linear accelerator. In the current study, models were generated and validation measurements were obtained for 6, 9, 12, 16, and 20 MeV for both the Varian iX and TrueBeam linear accelerators for 6 × 6, 10 × 10, 15 × 15, and 20 × 20 cm cones at SSDs of 100, 105, and 110 cm. This validation set covers essentially all possible electron treatments that may be encountered clinically.

For clinical treatments involving SSDs other than the modeled 100, 105, or 110 cm, it may be possible to determine the insert factor for an arbitrary SSD by using the insert factors calculated at the three modeled SSDs and interpolating for the insert factor at the desired SSD. Preliminary investigation showed the predicted insert factors follow a quadratic trend, from which a second order polynomial could be fit and used to calculate the insert factor at any SSD within the prediction space. Validation of this technique is a topic of future investigation.

One of the main advantages of the technique highlighted by Biggs is the ease of creating the model due to the relatively small number of measurements that are required. The spline model requires a minimum of eight empirical measurements, and the expected uncertainty of the technique to predict insert factors for a 12 MeV beam and 10 cm cone with this number of measurements was estimated as 0.5%.^17^ The results from the current study demonstrated a slightly larger overall uncertainty of 0.6%. This uncertainty may be more representative of the prediction accuracy of this technique over the wide range of energy, cone, and SSD combinations possible in electron treatments. Overall, this study found the suggested bounding shape method to be an efficient way of creating a model with high coverage. However, initial results of the current study showed larger disagreement in the model predictions for insert shapes that were in the middle of the parameter space when only using eight bounding shapes. Because of this, additional empirical measurements of shapes with dimensions in this parameter space were added to the models for the larger cones. This was found to increase the accuracy of the insert factor prediction.

## 5 CONCLUSION

A previously published technique for the analytical calculation of electron insert factors using a bivariate spline model generated from elliptically parameterized empirical measurements was implemented for Varian linear accelerators. A main advantage of the technique is the ability of models to generate accurate predictions using a minimal amount of input data, with accurate results obtained using only eight data points for the smallest cone and maximum of 11 data points for the larger cones. This study provides expanded validation on the efficacy of using the technique to predict insert factors over the range of electron energy, cone size, and SSD combinations that may be used clinically. Across the range of validation measurements performed, the accuracy of the predicted insert factors was on par with other proposed techniques in the literature, with an average absolute difference of 0.8%, maximum difference of 2.1%, and an uncertainty of 0.6%. Therefore, this methodology can be deemed acceptable for clinical use. It can be reliably used to predict insert factors for any Varian linear accelerator.

## CONFLICT OF INTEREST

The authors declare that there is no conflict of interest that could be perceived as prejudicing the impartiality of the research reported.

## AUTHOR CONTRIBUTION

Both the authors contributed equally to conception and design of the study, acquisition and analysis of the data, and final writing of the manuscript.

## Data Availability

The data that support the findings of this study are available from the corresponding author upon reasonable request.
